# *Moesziomyces aphidis* Bloodstream Infection in Oncologic Patient: First Report in Poland

**DOI:** 10.3390/jof11020095

**Published:** 2025-01-24

**Authors:** Beata Sulik-Tyszka, Jolanta Małyszko, Agnieszka Pęczuła, Sylwia Jarzynka

**Affiliations:** 1 Department of Microbiology, Maria Sklodowska-Curie National Research Institute of Oncology, 02-781 Warsaw, Poland; 2 Laboratory of Microbiology, University Center of Laboratory Medicine, Medical University of Warsaw, 02-091 Warsaw, Poland; 3 Department of Nephrology, Dialysis and Internal Medicine, Medical University of Warsaw, 02-091 Warsaw, Poland; 4 Department of Gastroenterology and Internal Medicine, Medical University of Warsaw, 02-091 Warsaw, Poland; 5 Department of Medical Biology, Medical University of Warsaw, 02-091 Warsaw, Poland

**Keywords:** *Moesziomyces* (*Pseudozyma*) spp., fungal bloodstream infections, rare yeast, antifungal resistance

## Abstract

*Moesziomyces* spp. (*Pseudozyma*) is a genus recognized as a new opportunistic human pathogen, causing systemic infections including premature neonates and adult patients. These fungi’s natural resistance to caspofungin enables them to spread through vascular catheter colonization, making them a new etiological agent associated with fungal bloodstream infections (FBIs) and a significant contributor to high mortality rates. In this report, we present a case of fungemia caused by *Moesziomyces aphidis* species in a patient with medical history that revealed pancreatic cancer infiltrating the duodenum and bile ducts. During hospitalization, the *M. aphidis* was cultured twice from peripheral blood samples on Sabouraud agar. The strain was sensitive to amphotericin B and voriconazole. In vitro susceptibility testing revealed resistance to fluconazole, caspofungin, anidulafungin, and micafungin. Antifungal therapy with voriconazole resulted in the resolution of clinical symptoms associated with fungal infection. Related to *M. aphidis* fungemia, we reviewed a total of three cases in Europe published in the PubMed database between 2003 and 2024. To the best of our knowledge, this is the first case of *M. aphidis* FBI in Poland and the fourth case in an adult patient in Europe.

## 1. Introduction

In the XXI century, knowledge about fungi was quite extensive, yet it was insufficient for effectively combatting these pathogens. In recent years, there has been an increase in fungal infections caused by species previously considered solely saprophytic. Thanks to the synthesis of new antifungal drugs, primarily echinocandins, we have new antifungal agents. However, fungal infections still pose a significant diagnostic and therapeutic challenge [1,2]. The most common infectious agent of candidemia is *Candida albicans*. However, in recent years, there has been an observed increase in the prevalence of other *Candida* species with varying resistance profiles. This trend is associated with the natural selection of species. Antifungal prophylaxis has led to a rise in strains with inherent resistance to fluconazole (*Candida krusei*) or reduced susceptibility to azoles (*Candida glabrata*, *Candida inconspicua*, *Candida norvegensis*). From 2 to 3%, the etiologic factor of fungal sepsis is attributed to molds from the *Aspergillus* genus [3,4]. Other species occur very rarely [5]. However, in the last 40 years, there has been a significant increase in opportunistic fungi causing high-mortality catheter-related fungal bloodstream infections (FBIs), associated with vascular catheter colonization. Both well-known and newly identified species of opportunistic fungi are exhibiting an increasing number of virulence factors that contribute to antifungal drug resistance, including the ability to form biofilms, genetic mutations, efflux pumps, and reduced susceptibility because of drug exposure. An example is *Candida auris*, which poses a serious threat to global public health due to its worldwide distribution, multidrug resistance, and high mortality [6]. The seriousness of this phenomenon was noticed by the World Health Organization, which in 2022 issued a fungal priority pathogens list [7]. The document covers critical fungal pathogens such as the yeast-like *C. auris*, *C. albicans*, *Cryptococcus neoformans,* and the mold *Aspergillus fumigatus*. The report also mentions some rare fungal species.

Our report focuses on a case of a rare infection caused by the species *Moesziomyces aphidis*. In the *Basidiomycetes* group, a new genus called *Moesziomyces* (*syn. Pseudozyma, syn. Dirkmeia*) emerged in 2003 in Thailand [8], which is currently identified as an opportunistic human pathogen, including systemic infections. Currently, over ten species have been identified within this genus, with four (*M. aphidis*, *M. antarcticus*, *M. parantarctica, M. bullatus*) being recognized as the main opportunistic adult and premature neonate fungal pathogens naturally resistant to caspofungin [9,10,11]. Due to drug resistance, this pathogen is gaining greater clinical significance. Globally, 10 cases have been reported so far. It is a very rare infectious factor in fungal infections [12]. FBIs caused by this genus have been predominantly reported in countries across Asia (Thailand, China, India, Korea), in South America (Brazil, Argentina), and Africa (Nigeria). In Europe, only four cases have been recorded, in France [9], Italy [11], Greece [12], and, as with this study, in Poland. Herein, we report to the best of our knowledge the first case of *M. aphidis* fungemia in an adult patient in Poland and the fourth case in Europe.

## 2. Case Report

### 2.1. Description of Patients

An 88-year-old patient was hospitalized due to obstructive jaundice at the Department of Nephrology, Dialysis and Internal Medicine, University Clinical Center of the Warsaw Medical University. All data were analyzed during a routine diagnosis of fungemia.

**Figure 1:**
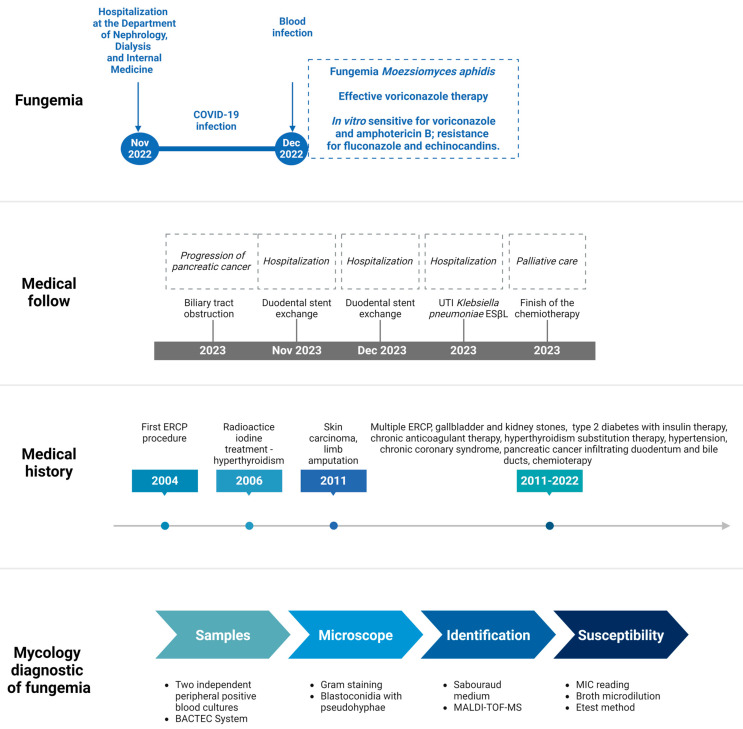
Medical history and fungemia diagnostic timeline between 2004 and 2023. Created in https://BioRender.com.

Medical history revealed pancreatic cancer infiltrating the duodenum and bile ducts with chemotherapy treatment (Figure 1) and confirmed previous procedures of cholecystectomy due to gallbladder stones and the repair of a right inguinal hernia. The patient also had a history of kidney stones. Endoscopic retrograde cholangiopancreatography (ERCP) with stent placement in the bile ducts was performed multiple times (between 2004 and 2023). In the patient’s medical history, type 2 diabetes treated with insulin was noted. The patient was on chronic anticoagulant therapy with Apixaban and had a history of radioactive iodine treatment for hyperthyroidism (in 2006). Hypothyroidism was managed with substitution therapy. Additionally, the patient underwent left lower limb amputation (at the thigh level) in 2011 due to extensive squamous cell carcinoma of the skin and pathological fracture resulting from bone and marrow infection. The patient was taking the following medications: insulin, levothyroxinum natricum, metoprolol, perindopril, eplerenone, potassium supplements, furosemide, apixaban, simvastatin, finasteride, pancreatinum, and pantoprazole.

In November 2022, the patient was diagnosed with a COVID-19 infection and hospitalized. PCR testing and antigen tests were performed due to the suspicion of a COVID-19 infection. The risk factors for infection included the patient’s cancer, multiple surgical procedures, type 2 diabetes, and COVID-19 infection. During the physical examination, general wasting of the patient was observed. There were no signs of meningeal symptoms. Other medical conditions included essential hypertension, chronic coronary syndrome, and paroxysmal atrial fibrillation. During hospitalization in December 2022, the patient was diagnosed with a blood infection of fungal etiology. Blood cultures were taken from the patient at the suspicion of fungemia, and no other microorganisms were cultured except for fungi (Figure 1). The patient was observed to have persistent fever of undetermined etiology despite the use of broad-spectrum antibiotics and negative blood cultures for bacteria. On the fourth day of incubation the yeast-like fungi were cultured from the blood. Despite the diagnosis of invasive fungal infection and the administration on effective antifungal therapy with voriconazole (400 mg twice daily initially, followed by 200 mg twice daily) over 14 days via oral administration, the oncology patient, who was in relatively good condition, was discharged from the hospital and referred to oncologic outpatient care.

Progression of pancreatic cancer infiltrating the duodenum and bile ducts was observed. During another hospitalization in 2023, the patient underwent both analytical and imaging examinations. In basic laboratory tests, the total bilirubin level was 8.82 mg/dL, showing signs of cholestasis, elevated C-reactive protein (CRP) at 68 mg/dL, and a decreased hemoglobin level at 8.1 g/dL. Due to biliary tract obstruction, a duodenal stent exchange was performed in November 2023, and the next and last time this same procedure was conducted was in December 2023. After the operative procedure, the *Klebsiella pneumoniae* ESβL urinary tract infection was diagnosed and treated by antibiotics. In the postoperative period, general parameters deteriorated. The patient’s general condition deteriorated, and due to progression of the oncological disease, chemotherapy was discontinued, and palliative care was initiated.

### 2.2. Mycological Investigation

**Figure 2:**
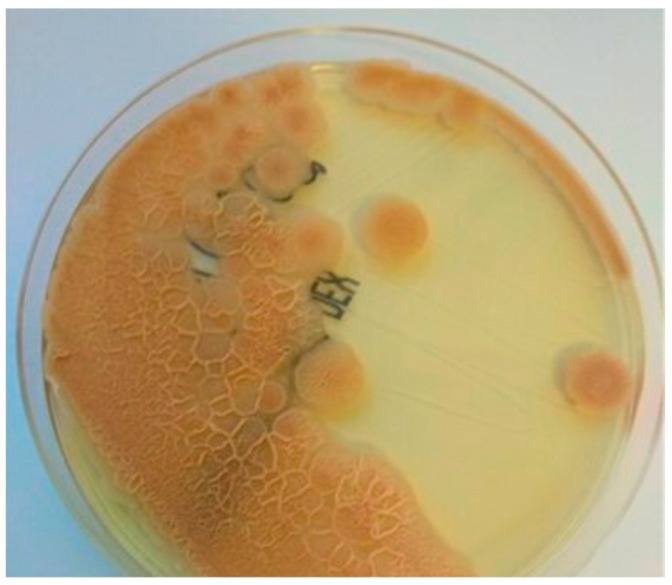
*M. aphidis* colonies—24 h culture on selective Sabouraud medium. Morphology growth: *M. aphidis* had a regular colony morphology: initially smooth and creamy in appearance. Over the course of 5 days of incubation, the colonies exhibited morphological changes, becoming wrinkled in texture and transitioning in color to orange. Author: Beata Sulik-Tyszka.

During hospitalization, the patient was confirmed to have fungemia caused by *Moesziomyces aphidis*. *M. aphidis* was cultured twice from peripheral blood samples from two independent injections on BD BACTEC™ Mycosis IC/F culture vials and a BACTEC™ blood culture instrument (BD Diagnostic Systems; Sparks, MD, USA) on the fourth day of incubation and then on Sabouraud agar with gentamicin and chloramphenicol (BioMaxima, Lublin, Poland), (Figure 2). Cultures were incubated at 30 °C under aerobic conditions. Direct microscopic examination using the Gram staining technique revealed blastoconidia with branched pseudohyphae. A direct stained preparation from the positive blood sample was made on an emergency basis and was not preserved in the collection. Species identification was confirmed using matrix-assisted laser desorption/ionization time-of-flight mass spectrometry (MALDI-TOF) with MALDI Biotyper Sirius (Bruker Daltonik, Bremen, Germany). The susceptibility was tested quantitatively with an MIC (minimal inhibitory concentration) reading using the colorimetric broth microdilution method on a Sensititre Aris System (YeastONE MIC Plate with Micafungin, Thermo Fisher Scientific, Cleveland, OH, USA) and the Etest technique (bioMérieux, Warsaw, Polska). The strain exhibited low MIC values against amphotericin B (0.125 µg/mL) and voriconazole (0.125 µg/mL) for both methods. However, in vitro susceptibility testing showed resistance of the *M. aphidis* strain to fluconazole (>256 µg/mL, both methods), caspofungin (>32 µg/mL E-test, >8 µg/mL Aris), anidulafungin (>32 µg/mL E-test, >8 µg/mL Aris), and micafungin (>32 µg/mL E-test, >8 µg/mL Aris) (Table 1). The results were interpreted according to the EUCAST guidelines from 2022 [13], which were applicable during the year when the fungemia occurred, as well as according to the new guidelines from 2023 [14] and the 2024 breakpoint tables for common fungi [15,16] and for rare fungi [17]. In both guidelines, the MICs for amphotericin B and voriconazole were unchanged. Changes in the breakpoint’s values were demonstrated only for echinocandins to which the strain *M. aphidis* was resistant (i.e., they exhibited MICs higher than the maximum drug concentration). During the selection of antifungal therapy, the interpretation from EUCAST 2022 was applied. The results and interpretation of the susceptibility of *M. aphidis* strains described by other authors, including the European cases presented in Table 1 [9,11,12] and cases of another rare yeast and mold [18], were also highly significant for the treatment method of the Polish case.

In 2024, new recommendations for the interpretation of MICs for rare fungi were published, but they do not refer to the species *M. aphids*. However, in the current assessment of drug susceptibility results, these guidelines were also included in the analysis. Additionally, this is the fourth case described in Europe, and therefore, there is a lack of definitive data on therapy. In this case, we describe the in vitro resistance of the strain to echinocandins, and fluconazole suggests that these drugs should not be used in therapy due to their very high MIC values. For amphotericin B, the MIC value of 0.125 µg/mL was very low, and similarly, voriconazole showed favorable results. These drugs are recommended for therapy despite the absence of clinical interpretation according to EUCAST and CLSI (Clinical and Laboratory Standards Institute) guidelines [19]. Most fungemia are endogenous, and echinocandins are used as first-line therapy. In this case, anidulafungin, caspofungin, and micafungin with MIC values > 32 µg/mL, as well as fluconazole with a value > 256 µg/mL, should be considered in vitro resistant.

Effective antifungal therapy was initiated, leading to the resolution of clinical symptoms of fungal infection. The 14 days of therapy included oral administration of voriconazole at a dose of 800 mg/day (first day) followed by 400 mg/day, which was successful. The patient did not experience any side effects after the use of voriconazole in the therapy. There was no hepatic impairment nor any increase in bilirubin or transaminase levels with the therapy used. The subsequent blood cultures after treatment were negative.

## 3. Discussion

*M. aphidis* belongs to yeast-like fungi, saprophytic organisms commonly found in the environment. *M. aphidis* is known to infect a variety of plant species, including cucumbers, melons, peas, roses, rice, tomato, and many others. It spreads through airborne spores and thrives in warm, dry conditions. It inhabits leaves, flowers, soil, and may participate in the decomposition of leaves and branches. Powdery mildew can weaken plants, reduce yields in crops, and diminish the esthetic value of ornamental plants [10]. Fungi from the genus *Moesziomyces* belong to the *Basidiomycota* type, *Ustilaginomycota* subphylum, *Ustilaginomycetes* class, and *Ustilaginales* order [20]. The pathogenicity of this species is low; however, in individuals with compromised immune systems or those using immunosuppressive drugs, it can lead to subcutaneous infections and disseminated infections [12,21].

A literature review of the cases associated with *P. aphidis* indicates a total number of 56 articles associated with keywords “*Pseudozyma aphidis*” between 2003 and 2024, which were collected from the PubMed database (http://www.ncbi.nlm.nih.gov/pubmed, access on 7 January 2025). In most publications, *M. aphidis* has been described as a factor of plant infections and as a source of metabolites with biological activity, e.g., in the biodegradation process. Only 12 publications were associated with infection caused by *P. aphidis*, nine fungemia in adults or neonates [8,9,10,11,12,22,23,24,25] and three clinical cases of pulmonary infection [26], endophthalmitis [27], and leg mycoses [28]. In addition to the keyword results, another seven studies were independently found to describe “*Pseudozyma infection*”: BSI in stem cell transplant (SCT) recipient [29], CVC-related BSI [30], cardiovascular implantable electronic device (CIED) [31], skin mycosis [32], brain abscess [33], colonization of the respiratory tract [34], and one other [35].

Authors of publications with a global scope emphasize that *Pseudozyma* spp. was first described as infectious agent in 2003, based on blood culture isolates from three patients in Thailand. The isolated strains were classified as *P. antarctica*, *P. parantarctica*, and *P. thailandica* [8]. In Europe, FBIs caused by *Pseudozyma aphidis* have been described only three times so far [9,11,12]. The clinical case described in our original work represents the fourth diagnosed case of invasive infection caused by *M. aphidis* in European countries.

Risk factors for fungal infections include the following: congenital immunodeficiency, prolonged (>10 days) significant granulocytopenia (<0.5 × 10^9^/L), allogeneic stem cell transplantation, treatment with immunosuppressive drugs or corticosteroids, the use of central and dialysis catheters, and prior wide-spectrum antibiotic therapy, as well as hematologic malignancies and solid tumors [5,12]. In cases of fungemia caused by *M. aphidis*, among the results of global studies, the most important risk factors include the following: age, neutropenia (<3000 cells/µL), the presence of a central venous catheter (CVC), chronic kidney disease, thrombocytopenia, and previous bacteremia [9,10,11,12,24]. The clinical case presented in our study illustrates a patient with multiple risk factors for the development of invasive fungal infection, including a terminal-stage malignancy, numerous surgical procedures, broad-spectrum antibiotic therapy, and diabetes. The risk factors for infection also included a COVID-19 infection. In the post-pandemic period, numerous cases of rare fungal infections have been reported in patients who had recovered from COVID-19 [14,36,37,38,39,40,41,42,43,44,45]. The steroid therapy used in the treatment of COVID-19 might play a significant role in the development of the fungal infection caused by *M. aphidis*.

Patients with compromised hematologic status are particularly susceptible to infections caused by species belonging to the genus *Moesziomyces*. Hyonsoo Joo and colleagues recently reported a case of an immunocompromised patient with *M. aphidis* infection. A 51-year-old male with acute myeloid leukemia (AML) developed an *M. aphidis* fungemia with invasive fungal pneumonia during re-induction of the chemotherapy. After antifungal therapy, the patient’s condition improved [22]. Linn et al., in 2008, documented the first pediatric case of an FBI related to a CVC caused by *M. aphidis* in a 7-year-old female with short gut syndrome [23]. On the other hand, Prakash et al. described in 2014 the first case of *M. aphidis* fungemia in a newborn with low birth weight [24].

The first European case of an *M. aphidis* FBI in an adult was documented in 2015 by the French researcher Herb and colleagues in a patient after abdominal surgery [9]. The second case of this fungemia in an adult was recorded by Maccaro et al. in 2021 in Italy, in an intravenous drug-using woman who was newly diagnosed with aggressive lymphoma and subsequently developed a bloodstream infection [11]. In all the cases described above, pathogens of *Pseudozyma* spp. showed sensitivity to amphotericin B and selected azoles, while demonstrating in vitro resistance to echinocandins, fluconazole, and flucytosine [9,11]. This was confirmed in our case report. On the other hand, the Greek researchers Mpakosi et al. in 2022 described fungemia of this etiology in a newborn with severe morbidity and prolonged hospital stay, for the first time in Europe. In this case report, drug susceptibility tests did not confirm in vitro resistance to fluconazole. Clinical symptoms of the FBI only resolved after liposomal amphotericin B therapy was initiated. In the described global cases, amphotericin B was most commonly used in the treatment of *M. aphidis* infections, followed by oral voriconazole [12]. This confirms our case report, where the antifungal activity of amphotericin B and voriconazole was demonstrated with low MIC values at 0.125 µg/mL. Ultimately, therapy with voriconazole led to an improvement in the patient’s clinical condition. In vitro resistance to fluconazole was also demonstrated. We summarize the available results of in vitro susceptibility testing against *Moesziomyces aphidis* isolated from European fungemia [9,11,12] in Table 1. The antifungal agents tested frequently were amphotericin B, azoles, and echinocandins.

The results emphasize the importance of determining the drug susceptibility of *M. aphidis* based on MIC values, enabling targeted therapy in line with recommendations for the treatment of fungal infections [13,14,15,16,17]. Antifungal therapy remains a significant challenge for clinicians, especially in the case of rare fungal infections. The lack of standardized susceptibility testing for rare yeasts according to guidelines from the Infectious Diseases Society of America (IDSA) means that appropriate treatment can only be applied based on case reports and expert recommendations. The few available recommendations suggest the European Confederation of Medical Mycology (ECMM) in cooperation with the International Society for Human and Animal Mycology (ISHAM), the American Society for Microbiology (ASM), and the European Society of Clinical Microbiology and Infectious Diseases (ESCMID) [46,47,48,49].

It is worth noting that when using commercially available systems in routine diagnostic laboratories, it is not possible to identify this drug-resistant pathogen. Sequencing and phylogenetic analysis can aid in the direct detection of isolates from blood or tissues. The taxonomy of yeast-like fungi is constantly evolving, making the species identification of rare fungal infections challenging [22]. However, it is important to remember that in routine sepsis diagnostics, the “gold standard” still involves obtaining material from the site of infection and rapidly identifying the pathogen using matrix-assisted laser desorption/ionization time-of-flight mass spectrometry (MALDI-TOF MS), as well as determining the strain’s susceptibility through microdilution methods. These methods are available in diagnostic laboratories and provide an accuracy comparable to nucleic acid sequencing. These methods are easy to implement and use in the routine diagnostics of fungemia caused by common and rare fungal species [6,50,51,52,53,54,55]. The assay based on utilize characteristic protein patterns to reliably and precisely identify specific microorganisms by matching the appropriate pattern in an extensive reference library, which is continuously updated. These databases are available both for research use only (RUO) and for in vitro diagnostic (IVD) purposes. Currently, the identification capabilities of the system exceed 2800 species of bacteria and fungi. MALDI-TOF MS is based on multiple measurements of a single, defined strain to capture the biological variability of the organism. Conventional phenotypic methods of pathogen identification, including colony morphology assessment and microscopic examination, are often insufficient. Available biochemical identification tests cannot detect the etiological agent of rare fungemia. Only genetic methods and the MALDI-TOF mass spectrometry system can accurately identify rare fungal species. The introduction of modern diagnostic methods increases the chances of patient survival. The clinical significance of rare cases and reliable species identification based on well-established diagnostic databases allows for accurate pathogen identification [2,12,56,57,58]. In the study by Prakash et al., in vitro sensitivity of *M. aphidis* to amphotericin B, voriconazole, isavuconazole, and posaconazole was demonstrated. High MIC values were observed for fluconazole and echinocandins. Amphotericin B was used in therapy. The transmission route of the pathogen could not be established. It could have originated from the mother’s urogenital tract or from nosocomial infection [24]. In the treatment of *M. aphidis* in the present study, amphotericin B and voriconazole were therapeutic options for patients with this infection due to their low MIC values. The high MIC values of echinocandins determined by the microdilution method in our case, as well as in the literature reports, may indicate a lack of activity of these drugs. This could be attributed to the low levels of (1,3)-β-D-glucan in the cell wall of *M. aphidis*. The high MIC values of fluconazole confirm the lack of activity of this drug in the described case.

The introduction of rapid diagnostic methods is essential in clinical microbiology and plays a crucial role in the implementation of effective and timely antifungal therapy, particularly in cancer patients.

In cases of rare-yeast infections, an interdisciplinary approach allows for faster diagnosis, which is especially important in oncological immunocompromised patients with a high risk of invasive fungal infections. In these clinical groups, mycological diagnostic algorithms should combine conventional tests and new approaches to improve fungal genus identification and strain susceptibility and support effective antifungal therapy.

## 4. Conclusions

*Moesziomyces aphidis* is an opportunistic human pathogen causing systemic infections, particularly in patients with underlying conditions. This case report highlights the first documented occurrence of *M. aphidis* fungemia in Poland and the fourth in an adult patient in Europe. The isolate demonstrated resistance to multiple antifungal agents, including fluconazole and echinocandins, but was susceptible to amphotericin B and voriconazole. Successful treatment with voriconazole indicates its potential effectiveness against *M. aphidis* infections. This case highlights the need for increased awareness and further research into the clinical management of bloodstream infections caused by this rare but significant fungal pathogen.

## Figures and Tables

**Table 1:** Antifungal susceptibility of *Moesziomyces aphidis* isolate from blood samples.

Antifungal Agent	MIC Range Value (µg/mL) of Our Isolate	Range of the MIC Value (µg/mL) from European Cases
amphotericin B	0.125 ^a,b^	0.19 ^c^; 0.5 ^d^; 0.25 ^e^
voriconazole	0.125 ^a,b^	0.032 ^c^; 0.031 ^d^; 0.015/0.006 ^e^
itraconazole	ND	0.19 ^c^; 0.5 ^d^; 0.03/0.06 ^e^
fluconazole	>256 ^a,b^	16 ^c^; 4 ^d^; 2/4 ^e^
isavuconazole	ND	ND ^c,d,e^
posaconazole	ND	0.094 ^c^; 0.031 ^d^; 0.03/0.125 ^e^
flucytosine	ND	>32 ^c^; ND ^d^; >64 ^e^
caspofungin	>8 ^a^; >32 ^b^	>32 ^c^; >8 ^d^; >8 ^e^
anidulafungin	>8 ^a^; >32 ^b^	ND ^c^; >8 ^d^; >8 ^e^
micafungin	>8 ^a^; >32 ^b^	ND ^c^; >8 ^d^; >8 ^e^

^a^ Colorimetric broth microdilution Sensititre Aris System (YeastONE MIC Plate with Micafungin, Thermo Fisher Scientific, Cleveland, OH, USA); ^b^ Etest technique (bioMérieux, Warsaw, Polska); ^c^ Etest technique (BioMérieux, Lyon, France) [9]; ^d^ microdilution (Micronaut, Bruker, Milano, Italy) [11]; ^e^ colorimetric broth microdilution Sensititre Aris System YeastONE (Thermo Fisher Scientific, Cleveland, OH, USA), 72 h at 30 °C; /—results for two different controls’ growth [12]; ND—not detected.

## Data Availability

The original contributions presented in this study are included in the article. Further inquiries can be directed to the corresponding author.
